# *NR3C1* gene polymorphisms are associated with high-altitude pulmonary edema in Han Chinese

**DOI:** 10.1186/s40101-019-0194-1

**Published:** 2019-04-18

**Authors:** Yingzhong Yang, Hui Du, Yuhong Li, Wei Guan, Feng Tang, Qin Ga, Ri-Li Ge

**Affiliations:** 1 Research Center for High Altitude Medical Sciences, School of Medicine, Qinghai University, 16 Kunlun Rd, Xining, 810001 Qinghai People’s Republic of China; 2 Basic and Applied Key Laboratory for High Altitude Medical Science and Technology of Qinghai, Xining, 810001 Qinghai China; 3 Qinghai-Utah United Key Laboratory for High Altitude Medical Science, Xining, 810001 Qinghai China; 4 Department of Respiration, Affiliated Hospital, Qinghai University, Xining, 810001 Qinghai China

**Keywords:** HAPE, *NR3C1*, Polymorphism, Susceptibility

## Abstract

**Background:**

High-altitude pulmonary edema (HAPE) is a life-threatening form of non-cardiogenic edema which occurs in unacclimatized individuals after rapid ascent to high altitude. *NR3C1* gene encodes for glucocorticoid receptor (GR) which plays an important role in stress and inflammation. This study aimed to investigate the association of *NR3C1* polymorphisms with the susceptibility to HAPE in Han Chinese.

**Methods:**

The 30 SNPs in the *NR3C1* gene were genotyped by the Sequenom MassARRAY SNP assay in 133 HAPE patients (HAPE-p) and 135 matched Han Chinese resistant to HAPE (HAPE-r). The genotypic and allele frequencies, odds ratios (ORs), and 95% confidence intervals (95% CIs) were calculated, respectively.

**Results:**

The 12 SNPs showed a significant difference between the HAPE-p and HAPE-r groups. In allelic model analysis, we found that the allele “A” of rs17287745, rs17209237, rs17209251, rs6877893, and rs1866388; the allele “C” of rs6191, rs6188, and rs2918417; the allele “T” of rs33388 and rs4634384; and the allele “G” of rs41423247 and rs10052957 were associated with increased the risk of HAPE. In the genetic model analysis, we found that rs17287745, rs6191, rs6188, rs33388, rs2918417, rs6877893, rs1866388, rs41423247, rs4634384, and rs10052957 were relevant to the increased HAPE risk under the dominant model.

In addition, the haplotype AACACTCAAGTG of the 12 SNPs was detected to be significantly associated with HAPE risk (OR = 2.044, 95%CI = 1.339~3.120, *P* = 0.0008), while the haplotype GGAGCACGACCG was associated with the decreased risk of HAPE (OR = 0.573, 95% CI = 0.333~0.985, *P* = 0.0422).

**Conclusions:**

Our findings provide new evidence for the association between SNPs in *NR3C1* and an increased risk of HAPE in the Chinese population. *NR3C1* polymorphisms are associated with the susceptibility to HAPE in Han Chinese.

**Electronic supplementary material:**

The online version of this article (10.1186/s40101-019-0194-1) contains supplementary material, which is available to authorized users.

## Background

High-altitude pulmonary edema (HAPE) is non-cardiogenic pulmonary edema that usually occurs at altitudes above 2500 m in rapidly ascending non-acclimatized individuals within the first week after arrival [1, 2]. HAPE is characterized by high pressure in pulmonary arteries, with edema in pulmonary interstitial tissue and alveoli, leading to pulmonary capillary stress failure and a high permeability type of edema. HAPE was defined as a non-inflammatory hemorrhagic pulmonary edema, which may evolve with the features of secondary inflammation [3]. Circulating inflammatory markers of IL-6, IL-1ra, and CRP are upregulated in response to high altitude, and hypoxia-induced inflammation at high altitude may contribute to the development of HAPE [4, 5].

The incidence of HAPE could be reduced by dexamethasone [6]. Glucocorticoids (GCs) have various effects, which are exclusively mediated by the intracellular glucocorticoid receptor (GR). After the binding to GCs, GR is translocated to the nucleus where it interacts with glucocorticoid-responsive elements of different genes to drive gene transcription [7]. In inflammatory reactions, GC inhibits the production of pro-inflammatory cytokines and stimulates the production of anti-inflammatory cytokines [8]. *NR3C1* (nuclear receptor subfamily 3, group C, member 1) gene is located on chromosome 5q31-q32 and encodes for the human GR [9]. Variants in the *NR3C1* gene may contribute to the spectrum of GC responses in different diseases [7, 10–12]. Previously, we screened the variants in exons of the *NR3C1* gene and reported that rs6194 polymorphism was correlated with HAPE susceptibility [12]. In this study, we aimed to investigate the association of the polymorphisms in non-coding regions of the *NR3C1* gene with the susceptibility to HAPE in Han Chinese.

## Materials and methods

### Subjects

HAPE patients (HAPE-p) had been hospitalized in Yushu People’s Hospital from 2010 to 2017 owing to the onset of HAPE after arriving 1 to 7 days at Yushu (3760 m) of Qinghai province. The patients were diagnosed with HAPE based on the diagnostic criteria [13]. The blood samples and data were collected. Healthy lowlanders resistant to HAPE (HAPE-r) were enrolled from the same area, with matched age, gender, workplace, and type of work. These subjects had not suffered from HAPE or any other mountain sickness after staying at high altitude for at least 3 months. One hundred thirty-three HAPE and 135 matched HAPE-r were enrolled. This study was approved by the Ethics Committee of the Medical College of Qinghai University, and every subject signed written consent. All subjects were of lowlander Han Chinese ethnicity and had no blood relationship with any other enrolled subject.

### Clinical characteristics

Hemoglobin concentration, hematocrit, and percent oxygen saturation were determined from venous blood samples using the Mindray Hematology Analyzer (BC-2300, Shenzhen, People’s Republic of China) and the Pulse Oximeter (Ohmeda 3700 Pulse Oximeter, DatexOhmeda, Boulder, Colorado, USA). Sample collection and DNA extraction were the same with the previous one [12].

### SNP selection and genotyping

In this study, a total of 30 SNPs (minor allele frequencies (MAFs) > 5%) in the *NR3C1* gene have been identified in the HapMap Han Chinese population and are located in non-coding regions. SNPs were genotyped by the single-base extension detecting technology (iPLEX) (Capital Bio Corporation, Beijing, China). The primers for PCR and single-base extension were designed by using the Sequenom MassARRAY Assay Design Genotyping Software and Tools (Sequenom, San Diego, CA, USA). PCR was performed under the following thermal cycling conditions: 94 °C for 4 min, then 94 °C for 20 s, 56 °C for 30 s, and 72 °C for 1 min for 45 cycles, and 72 °C for 4 min. PCR products were treated with shrimp alkaline phosphatase to remove free deoxyribonucleoside triphosphates, and single-base extension reaction was performed in a system consisted of 2.0 mL of EXTEND MIX, 0.619 mL of ddH_2_O, 0.94 mL of Extend primer mix, 0.2 mL of iPLEX buffer plus, 0.2 mL of iPLEX terminator, and 0.041 mL of iPLEX enzyme (Sequenom, San Diego, CA, USA). The thermal cycling conditions were as follows: 94 °C for 30 s, then 94 °C for 5 s, 52 °C for 5 s, and 80 °C for 5 s for 40 cycles, and 72 °C for 3 min. The purified extension products were dispensed onto a 384-element SpectroCHIP bioarray (Sequenom, San Diego, CA, USA), and mass spectrometric analysis was performed using the MALDI-TOF (matrix-assisted laser desorption/ionization—time of flight) (Sequenom, San Diego, CA, USA). The results were analyzed using TYPER 4.0 software (Sequenom, San Diego, CA, USA).

### Statistical analysis

SPSS software (version 17.0, SPSS, Inc., Chicago, USA) was used for statistical analysis. Haplotype frequencies and the expected number of haplotypes for each individual were performed using SHEsis online software (http://analysis.bio-x.cn). Allele frequencies were calculated based on genotype frequencies in HAPE patients and control subjects, and inter-group differences were estimated by the chi-square test. Deviations from Hardy-Weinberg equilibrium (HWE) were assessed by the chi-square test. The criterion for significance was *P* < 0.05 for all comparisons.

## Results

### Basic characteristics of populations

The demographic and clinical characteristics of HAPE-p and HAPE-r are presented in Table 1. We found that percent oxygen saturation was significantly lower whereas heart rate was significantly higher in the HAPE-p group compared to that in the HAPE-r group. As expected, HGB and Hct were higher in the HAPE-r group compared to that in the HAPE-p group. There was no significant difference in age between HAPE-p and HAPE-r groups.

**Table 1:** High-altitude exposures and physiological phenotypes for the study populations

Groups	Subjects (*n*)	Gender	Altitude (m)	Average age (year)	HGB (g/dL)	Hct (%)	HR (b/m)	SPO_2_ (%)
HAPE-p	133	Male	3760	40.20 ± 9.91	157.24 ± 15.24*	47.79 ± 4.97*	109.73 ± 14.85^*^	62.46 ± 11.89*
HAPE-r	135	Male	3760	40.92 ± 5.15	172.80 ± 14.54	50.74 ± 8.15	80.84 ± 12.03	88.85 ± 4.17

Values are means ± SD. *P* < 0.05 vs HAPE-r

*HAPE-p* high-altitude pulmonary edema patients, *HAPE-r* high-altitude pulmonary edema resistant (control), *HGB* hemoglobin, *Hct* hematocrit, *HR* heart rate, *SPO*_*2*_ oxyhemoglobin saturation

### Genotype and allele distribution

We examined the genotypic distributions, allelic frequencies, and associations of 30 SNPs in all subjects. The SNP rs4244032 showed deviations from HWE, while other SNPs were in HWE in both groups (Table 2). The allele and genotype distributions of the 30 SNPs in the HAPE-p and HAPE-r groups are presented in Table 3 (Additional file 1). Twelve SNPs (rs17287745, rs17209237, rs6191, rs17209251, rs6188, rs33388, rs2918417, rs6877893, rs1866388, rs41423247, rs4634384, rs10052957) were significantly associated with HAPE (*P* < 0.05). In allelic model analysis, we found that the allele “A” of rs17287745, rs17209237, rs17209251, rs6877893, and rs1866388; the allele “C” of rs6191, rs6188, and rs2918417; the allele “T” of rs33388 and rs4634384; and the allele “G” of rs41423247 and rs10052957 were associated with increased the risk of HAPE. In the genetic model analysis, we found that rs17287745, rs6191, rs6188, rs33388, rs2918417, rs6877893, rs1866388, rs41423247, rs4634384, and rs10052957 were relevant to increased HAPE risk under the dominant model.

**Table 2:** SNPs information of the *NR3C1* gene and the Hardy–Weinberg Equilibrium (HWE) in the current population

SNP ID	Gene	Band	Position	Alleles A/B	Global MAF	*P* value	
						HAPE-p	HAPE-r
rs174048	*NR3C1*	5q31-q32	143270839	C/T	C = 0.1014/508	0.491	0.216
rs17287745	*NR3C1*	5q31-q32	143275450	G/A	G = 0.2528/1266	0.678	0.427
rs17209237	*NR3C1*	5q31-q32	143277647	G/A	G = 0.1542/772	0.543	0.394
rs6198	*NR3C1*	5q31-q32	143278056	C/T	C = 0.0839/420	0.965	0.826
rs6191	*NR3C1*	5q31-q32	143278591	A/C	A = 0.4016/2011	0.473	0.855
rs10482704	*NR3C1*	5q31-q32	143282198	A/C	A = 0.0080/40	1	0.966
rs258751	*NR3C1*	5q31-q32	143282715	A/G	A = 0.0547/274	0.491	0.216
rs17209251	*NR3C1*	5q31-q32	143289658	G/A	G = 0.1472/737	0.585	0.380
rs258813	*NR3C1*	5q31-q32	143295125	A/G	A = 0.2312/1158	0.323	0.082
rs6188	*NR3C1*	5q31-q32	143300779	A/C	A = 0.2306/1155	0.323	0.072
rs33388	*NR3C1*	5q31-q32	143317730	A/T	A = 0.3934/1970	0.473	0.759
rs33389	*NR3C1*	5q31-q32	143320934	T/C	T = 0.1064/533	0.491	0.236
rs2918417	*NR3C1*	5q31-q32	143346605	T/C	T = 0.2236/1120	0.321	0.072
rs6877893	*NR3C1*	5q31-q32	143347628	G/A	G = 0.4030/2018	0.320	0.952
rs10482642	*NR3C1*	5q31-q32	143348466	C/T	C = 0.0988/495	0.965	0.826
rs17399352	*NR3C1*	5q31-q32	143375125	C/T	C = 0.1394/698	0.798	0.272
rs2963155	*NR3C1*	5q31-q32	143376439	G/A	G = 0.2228/1116	0.553	0.075
rs2963156	*NR3C1*	5q31-q32	143378931	T/C	T = 0.1615/809	0.717	0.256
rs1866388	*NR3C1*	5q31-q32	143380220	G/A	G = 0.2139/1071	0.459	0.160
rs41423247	*NR3C1*	5q31-q32	143399010	C/G	C = 0.2546/1275	0.821	0.592
rs6189	*NR3C1*	5q31-q32	143400774	T/C	T = 0.0106/53	0.965	0.966
rs4634384	*NR3C1*	5q31-q32	143401132	C/T	C = 0.3972/1989	0.671	0.513
rs10052957	*NR3C1*	5q31-q32	143407136	A/G	A = 0.2212/1108	0.323	0.074
rs9324924	*NR3C1*	5q31-q32	143412919	T/G	T = 0.4808/2408	0.574	0.042
rs7701443	*NR3C1*	5q31-q32	143413085	A/G	G = 0.4605/2306	0.906	0.420
rs4244032	*NR3C1*	5q31-q32	143415160	G/A	G = 0.1326/664	0.000	0.002
rs4607376	*NR3C1*	5q31-q32	143416967	A/G	G = 0.3966/1986	0.782	0.724
rs12656106	*NR3C1*	5q31-q32	143429382	C/G	C = 0.3413/1709	0.840	0.429
rs12655166	*NR3C1*	5q31-q32	143429707	C/T		0.287	0.780
rs12521436	*NR3C1*	5q31-q32	143438042	A/G	A = 0.2776/1390	0.581	0.291

Alleles A/B = minor/major alleles, Global MAF = global minor allele frequency

*HAPE-p* high-altitude pulmonary edema patients, *HAPE-r* high-altitude pulmonary edema resistant (control)

**Table 3:** Comparison of genotype distributions and allele frequencies for SNPs associated with HAPE risk under the dominant and recessive model in both groups, respectively

SNP	Genotype/allele	HAPE-p (*n*%)	HAPE-r (*n*%)	OR (95% CI)	*χ* ^2^	*P*
rs17287745						
Genotype	AA	98 (73.7)	81 (60.0)			
	AG	33 (24.8)	45 (33.3)	1.650 (0.964–2.823)	3.365	0.067
	GG	2 (1.5)	9 (6.7)	5.444 (1.144–25.915)	5.558	*0.018*
Allele	A	229 (86.1)	207 (76.7)			
	G	37 (13.9)	63 (23.3)	1.884 (1.204–2.946)	7.841	*0.005*
Dominant model	AA	98 (73.7)	81 (60.0)			
	AG + GG	35 (26.3)	54 (40.0)	1.867 (1.113–3.131)	5.656	*0.017*
rs17209237						
Genotype	AA	95 (72)	83 (62.4)			
	AG	35 (26.5)	42 (31.6)	1.373 (0.803–2.349)	1.348	0.246
	GG	2 (1.5)	8 (6.0)	4.578 (0.946–22.166)	4.222	*0.040*
Allele	A	225 (85.2)	208 (78.2)			
	G	39 (14.8)	58 (21.8)	1.609 (1.028–2.517)	4.382	*0.036*
Dominant model	AA	95 (72.0)	83 (62.4)			
	AG + GG	37 (28.0)	50 (37.6)	1.547 (0.922–2.594)	2.748	0.097
rs6191						
Genotype	CC	88 (66.7)	69 (51.9)			
	CA	38 (28.8)	53 (39.8)	1.779 (1.055–2.999)	4.709	*0.03*
	AA	6 (4.5)	11 (8.3)	2.338 (0.824–6.638)	2.661	0.103
Allele	C	214 (81.1)	191 (71.8)			
	A	50 (18.9)	75 (28.2)	1.681 (1.118–2.526)	6.299	*0.012*
Dominant model	CC	88 (66.7)	69 (51.9)			
	CA + AA	44 (33.3)	64 (48.1)	1.855 (1.129–3.048)	5.999	*0.014*
rs17209251						
Genotype	AA	94 (72.3)	84 (62.7)			
	GA	34 (26.2)	42 (31.3)	1.382 (0.806–2.371)	1.388	0.239
	GG	2 (1.5)	8 (6.0)	4.476 (0.925–21.671)	4.079	*0.043*
Allele	A	222 (85.4)	210 (78.4)			
	G	38 (14.6)	58 (21.6)	1.614 (1.028–2.532)	4.380	*0.036*
Dominant model	AA	94 (72.3)	84 (62.7)			
	GA + GG	36 (27.7)	50 (37.3)	1.554 (0.924–2.614)	2.781	0.095
rs6188						
Genotype	CC	112 (84.2)	98 (73.1)			
	CA	21 (15.8)	36 (26.9)	1.959 (1.072–3.579)	4.877	*0.027*
	AA	0 (0.0)	0 (0.0)			
Allele	C	245 (92.1)	232 (86.6)			
	A	21 (7.9)	36 (13.4)	1.810 (1.027–3.193)	4.294	*0.038*
Dominant model	CC	112 (84.2)	98 (73.1)			
	CA + AA	21 (15.8)	36 (26.9)	1.959 (1.072–3.579)	4.877	*0.027*
rs33388						
Genotype	TT	88 (66.7)	69 (51.1)			
	AT	38 (28.8)	54 (40)	1.812 (1.076–3.052)	5.047	*0.025*
	AA	6 (4.5)	12 (8.9)	2.551 (0.911–7.141)	3.352	0.067
Allele	T	214 (81.1)	192 (71.1)			
	A	50 (18.9)	78 (28.9)	1.739 (1.160–2.607)	7.251	*0.007*
Dominant model	TT	88 (66.7)	69 (51.1)			
	AT+AA	44 (33.3)	66 (48.9)	1.913 (1.166–3.138)	6.666	*0.010*
rs2918417						
Genotype	CC	111 (84.1)	98 (73.1)			
	CT	21 (15.9)	36 (26.9)	1.942 (1.063–3.548)	4.741	*0.029*
	TT	0 (0.0)	0 (0.0)			
Allele	C	243 (92.0)	232 (86.6)			
	T	21 (8.0)	36 (13.4)	1.796 (1.018–3.167)	4.172	*0.041*
Dominant model	CC	111 (84.1)	98 (73.1)			
	CT + TT	21 (15.9)	36 (26.9)	1.942 (1.063–3.548)	4.741	*0.029*
rs6877893						
Genotype	AA	87 (68)	68 (51.1)			
	AG	35 (27.3)	54 (40.6)	1.974 (1.161–3.356)	6.385	*0.012*
	GG	6 (4.7)	11 (8.3)	2.346 (0.826–6.663)	2.678	0.102
Allele	A	209 (81.6)	190 (71.4)			
	G	47 (18.4)	76 (28.6)	1.779 (1.176–2.689)	7.553	*0.006*
Dominant model	AA	87 (68.0)	68 (51.1)			
	AG + GG	41 (32.0)	65 (48.9)	2.028 (1.226–3.356)	7.670	*0.006*
rs1866388						
Genotype	AA	116 (87.9)	105 (78.4)			
	GA	16 (12.1)	29 (21.6)	2.002 (1.030–3.894)	4.288	*0.038*
	GG	0 (0.0)	0 (0.0)			
Allele	A	248 (93.9)	239 (89.2)			
	G	16 (6.1)	29 (10.8)	1.881 (0.996–3.552)	3.892	*0.049*
Dominant model	AA	116 (87.9)	105 (78.4)			
	AG + GG	16 (12.1)	29 (21.6)	2.002 (1.030–3.894)	4.288	*0.038*
rs41423247						
Genotype	GG	93 (69.9)	76 (56.3)			
	CG	36 (27.1)	49 (36.3)	1.666 (0.984–2.819)	3.636	0.057
	CC	4 (3.0)	10 (7.4)	3.059 (0.923–10.142)	3.633	0.057
Allele	G	222 (83.5)	201 (74.4)			
	C	44 (16.5)	69 (25.6)	1.732(1.134–2.645)	6.544	*0.011*
Dominant model	GG	93 (69.9)	76 (56.3)			
	CG + CC	40 (30.1)	59 (43.7)	1.805 (1.091–2.985)	5.342	*0.021*
rs4634384						
Genotype	TT	87 (67.4)	68 (51.1)			
	CT	37 (28.7)	52 (39.1)	1.798 (1.061–3.047)	4.793	*0.029*
	CC	5 (3.9)	13 (9.8)	3.326 (1.131–9.786)	5.206	*0.023*
Allele	T	211 (81.8)	188 (70.7)			
	C	47 (18.2)	78 (29.3)	1.863 (1.234–2.812)	8.894	*0.003*
Dominant model	TT	87 (67.4)	68 (51.1)			
	CT + CC	42 (32.6)	65 (48.9)	1.980 (1.200–3.269)	7.214	*0.007*
rs10052957						
Genotype	GG	112 (84.2)	99 (73.3)			
	GA	21 (15.8)	36 (26.7)	1.939 (1.062–3.542)	4.734	*0.030*
	AA	0 (0.0)	0 (0.0)			
Allele	G	245 (92.1)	234 (86.7)			
	A	21 (7.9)	36 (13.3)	1.795 (1.018–3.165)	4.170	*0.041*
Dominant model	GG	112 (84.2)	99 (73.3)			
	GA + AA	21 (15.8)	36 (26.7)	1.939 (1.062–3.542)	4.734	*0.030*

Data are shown as odds ratio (OR), 95% confidence interval (CI), and *P* values comparing HAPE patients and control group

*P* value in italics indicates statistical significance after comparisons

*HAPE-p* high-altitude pulmonary edema patients, *HAPE-r* high-altitude pulmonary edema resistant (control)

### Linkage disequilibrium and haplotype analysis of the 12 SNPs

**Fig. 1:**
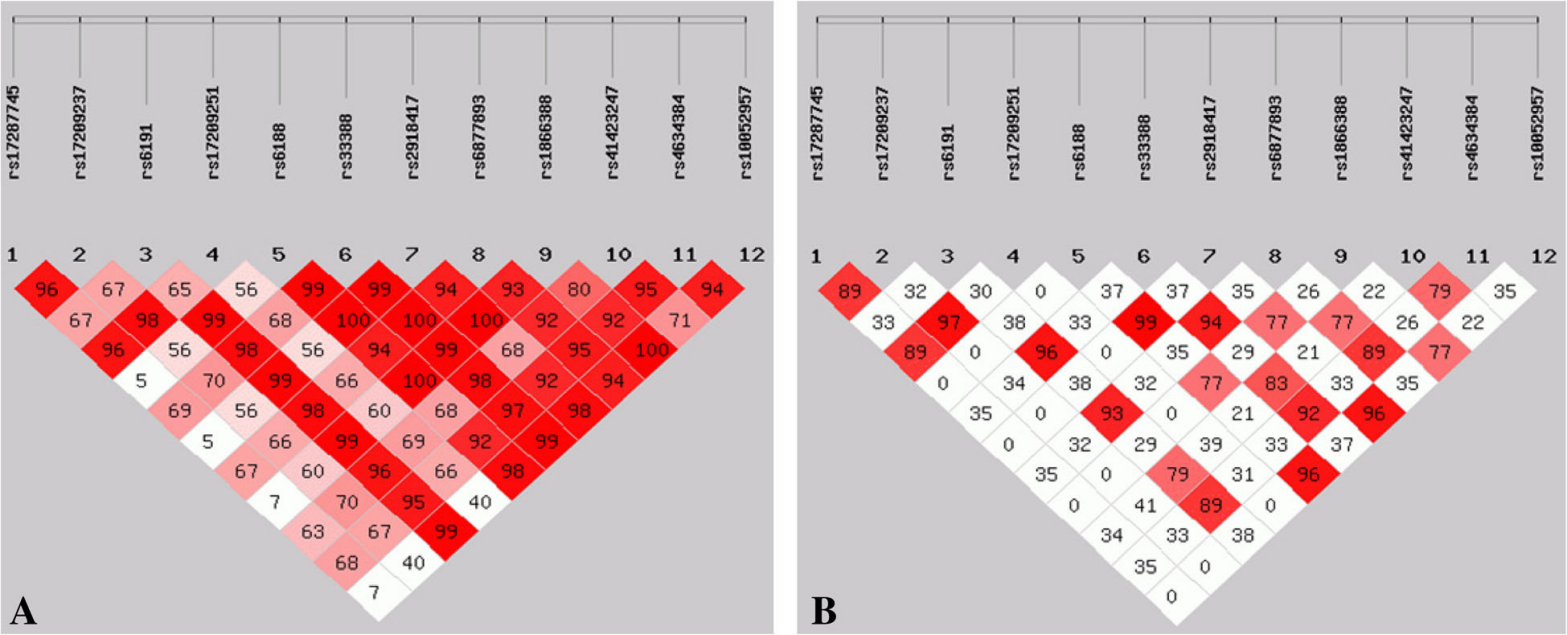
Linkage disequilibrium among the 12 SNPs of the *NR3C1* gene in the HAPE-p group (**a**) and HAPE-r group (**b**). The degree of genetic linkage between these SNPs is estimated as *D* values. Red panels indicate that there exists a strong pairwise linkage disequilibrium between adjacent SNPs whereas white panels indicate that the linkage disequilibrium is weak or non-existent

The locations of these SNPs are shown in Table 2, and the linkage disequilibrium of the SNPs is shown in Fig. 1. In the HAPE-p group, the SNPs were in strong linkage disequilibrium with each other (Fig. 1a). In comparison, few SNPs were found in linkage disequilibrium with each other in the control group (Fig. 1b). The degree of genetic linkage between these SNPs is estimated as *D* values. Red panels indicate that there exists a strong pairwise linkage disequilibrium between adjacent SNPs; the higher *D* value is represented with the darker red block, the higher degree of genetic linkage between the SNPs, whereas the white panels indicate that the linkage disequilibrium is weak or non-existent. The haplotype analysis of the 12 SNPs showed a significant difference between the two groups in Table 3, and the haplotype AACACTCAAGTG was detected to be significantly associated with HAPE risk (OR = 2.044, 95%CI = 1.339~3.120, *P* = 0.0008), while the haplotype GGAGCACGACCG was associated with decreased the risk of HAPE (OR = 0.573, 95% CI = 0.333~0.985, *P* = 0.0422) (Table 4).

**Table 4:** Haplotype analysis for 12 SNPs of the *NR3C1* gene in HAPE and control groups

Haplotype	HAPE-p (freq)	HAPE-r (freq)	*χ* ^2^	*P* value	Odds ratio [95%CI]
AAAAAATGGCCA	11.47 (0.043)	21.87 (0.081)	3.502	0.0613	0.500 [0.239~1.046]
AACACTCAAGTG	206.53 (0.776)	174.13 (0.645)	11.206	*0.0008*	2.044 [1.339~3.120]
GGAGCACGACCG	24.00 (0.090)	39.00 (0.144)	4.127	*0.0422*	0.573 [0.333~0.985]
GGCGCTCAAGTG	8.47 (0.032)	14.87 (0.055)	1.857	0.1729	0.553 [0.233~1.311]

*P* value in italics indicates statistical significance after comparisons, data are shown as odds ratio (OR), 95% confidence interval (CI)

*HAPE-p* high-altitude pulmonary edema patients, *HAPE-r* high-altitude pulmonary edema resistant (control), *χ*^*2*^ chi square

## Discussion

It is known that hypoxia can induce inflammation [14]. The levels of circulating proinflammatory markers were increased in healthy volunteers who spent three nights at an elevation higher than 3400 m [4]. In addition, HAPE patients at early stage showed increased counts of alveolar macrophages, neutrophils, and lymphocytes and markedly elevated concentrations of lactate dehydrogenase, IL-1beta, IL-6, IL-8, and TNF-alpha in the bronchoalveolar lavage fluid [15–17]. Moreover, vascular leakage, accumulations of inflammatory cells in multiple organs, and elevated serum levels of cytokines were observed in mice exposed to low oxygen concentration [18–20]. It has been speculated that hypoxia-induced inflammatory cytokines at high altitude may contribute to the development of HAPE by causing capillary leakage in the lung [4].

Oral administration of dexamethasone is effective in preventing acute mountain sickness (AMS) [6, 18]. Glucocorticoids are regarded as endogenous “dexamethasone” that regulate a broad spectrum of physiologic functions essential to the maintenance of basal and stress-related homeostasis, including inflammatory reactions. The effects of GCs are mediated by GRs, which are steroid/thyroid/retinoic acid nuclear receptor superfamily of transcription factors and function as a ligand-dependent transcription factor that regulates the expression of glucocorticoid-responsive genes. Approximately 20% of the genes expressed in human leukocytes are regulated by GCs [7]. Genetic mutations in *NR3C1* have been found to substantially diminish GR function [10]. Therefore, the genetic variations in the *NR3C1* gene are important to explain the pathogenesis of diseases.

In this study, we genotyped 30 SNPs of the *NR3C1* gene in Han Chinese with and without HAPE. For the first time, we reported significant differences between the two groups in 12 SNPs. Moreover, these polymorphisms were significantly associated with the risk of HAPE. The haplotype [AACACTCAAGTG] may increase the risk of HAPE. This new clue may better explain the genetic variations in the *NR3C1* gene contributing to the pathogenesis of HAPE.

## Conclusions

Our study suggests that the polymorphisms of *NR3C1* gene are associated with the susceptibility to HAPE in Han Chinese.

## Additional file

Additional file 1: **Table S1.** Comparison of genotype distributions and allele frequencies for SNPs associated with HAPE risk under the dominant and recessive model in both groups, respectively. (DOC 306 kb)
