# Supplementary material for: NR3C1 gene polymorphisms are associated with high-altitude pulmonary edema in Han Chinese
Source: J Physiol Anthropol. 2019 Apr 18;38:4. doi: 10.1186/s40101-019-0194-1 (PMC6472084; doi:10.1186/s40101-019-0194-1)
Supplement: Supplementary file 1 — Table S1. Comparison of genotype distributions and allele frequencies for SNPs associated with HAPE risk under the dominant and recessive model in both groups, respectively. (DOC 306 kb) [file 40101_2019_194_MOESM1_ESM.doc]

**Supplementary 1. Comparison of genotype distributions and allele frequencies for SNPs associated with HAPE risk under the dominant and recessive model in both groups, respectively**

| **SNP** | **Genotype/Allele** | **HAPE-p (n%)** | **HAPE-r (n%)** | **OR (95% CI)** | **X^2^** | **p** |
| --- | --- | --- | --- | --- | --- | --- |
| rs174048 |  |  |  |  |  |  |
| Genotype | TT | 118(88.7) | 109(80.7) |  |  |  |
|  | TC | 15(11.3) | 26(19.3) | 1.876(0.944-3.729) | 3.293 | 0.070 |
|  | CC | 0(0.0) | 0(0.0) |  |  |  |
| Allele | T | 251(94.4) | 244(90.4) |  |  |  |
|  | C | 15(5.6) | 26(9.6) | 1.783(0.922-3.448) | 3.021 | 0.082 |
| Dominant model | TT | 118(88.7) | 109(80.7) |  |  |  |
|  | TC+CC | 15(11.3) | 26(19.3) | 1.876(0.944-3.729) | 3.293 | 0.070 |
| rs6198 |  |  |  |  |  |  |
| Genotype | TT | 132(99.2) | 130(96.3) |  |  |  |
|  | TC | 1(0.8) | 5(3.7) | 5.077(0.585-44.053) | 2.667 | 0.103 |
|  | CC | 0(0.0) | 0(0.0) |  |  |  |
| Allele | T | 265(99.6) | 265(98.1) |  |  |  |
|  | C | 1(0.4) | 5(1.9) | 5.000(0.580-43.088) | 2.637 | 0.104 |
| Dominant model | TT | 132(99.2) | 130(96.3) |  |  |  |
|  | TC+CC | 1(0.8) | 5(3.7) | 5.077(0.585-44.053) | 2.667 | 0.103 |
| rs10482704 |  |  |  |  |  |  |
| Genotype | CC | 133(100.0) | 134(99.3) |  |  |  |
|  | CA | 0(0.0) | 1(0.7) | - | 0.989 | 0.320 |
|  | AA | 0(0.0) | 0(0.0) |  |  |  |
| Allele | C | 266(100.0) | 269(99.6) |  |  |  |
|  | A | 0(0.0) | 0(0.4) | - | 0.987 | 0.320 |
| Dominant model | CC | 133(100.0) | 134(99.3) |  |  |  |
|  | CA+AA | 0(0.0) | 1(0.7) | - | 0.989 | 0.320 |
| rs258751 |  |  |  |  |  |  |
| Genotype | GG | 118(88.7) | 109(80.7) |  |  |  |
|  | GA | 15(11.3) | 26(19.3) | 1.876(0.944-3.729) | 3.293 | 0.07 |
|  | AA | 0(0.0) | 0(0.0) |  |  |  |
| Allele | G | 251(94.4) | 244(90.4) |  |  |  |
|  | A | 15(5.6) | 26(9.6) | 1.783(0.922-3.448) | 3.021 | 0.082 |
| Dominant model | GG | 118(88.7) | 109(80.7) |  |  |  |
|  | GA+AA | 15(11.3) | 26(19.3) | 1.876(0.944-3.729) | 3.293 | 0.07 |
| rs258813 |  |  |  |  |  |  |
| Genotype | GG | 112(84.2) | 99(73.9) |  |  |  |
|  | GA | 21(15.8) | 35(26.1) | 1.886(1.030-3.452) | 4.297 | 0.038 |
|  | AA | 0(0.0) | 0(0.0) |  |  |  |
| Allele | G | 245(92.1) | 233(86.9) |  |  |  |
|  | A | 21(7.9) | 35(13.1) | 1.753(0.991-3.099) | 3.794 | 0.051 |
| Dominant model | GG | 112(84.2) | 99(73.9) |  |  |  |
|  | GA+AA | 21(15.8) | 35(26.1) | 1.886(1.030-3.452) | 4.297 | 0.038 |
| rs33389 |  |  |  |  |  |  |
| Genotype | CC | 118(88.7) | 110(81.5) |  |  |  |
|  | CT | 15(11.3) | 25(18.5) | 1.788(0.896-3.567) | 2.766 | 0.096 |
|  | TT | 0(0.0) | 0(0.0) |  |  |  |
| Allele | C | 251(94.4) | 245(90.7) |  |  |  |
|  | T | 15(5.6) | 25(9.3) | 1.707(0.879-3.317) | 2.543 | 0.111 |
| Dominant model | CC | 118(88.7) | 110(81.5) |  |  |  |
|  | CT+TT | 15(11.3) | 25(18.5) | 1.788(0.896-3.567) | 2.766 | 0.096 |
| rs10482642 |  |  |  |  |  |  |
| Genotype | TT | 132(99.2) | 130(96.3) |  |  |  |
|  | TC | 1(0.8) | 5(3.7) | 5.077(0.585~44.053) | 2.667 | 0.103 |
|  | CC | 0(0.0) | 0(0.0) |  |  |  |
| Allele | T | 265(99.6) | 265(98.1) |  |  |  |
|  | C | 1(0.4) | 5(1.9) | 5.000(0.580~43.088) | 2.637 | 0.104 |
| Dominant model | TT | 132(99.2) | 130(96.3) | - | - | - |
|  | TC+CC | 1(0.8) | 5(3.7) | - | - | - |
| rs17399352 |  |  |  |  |  |  |
| Genotype | TT | 104(78.8) | 96(72.2) |  |  |  |
|  | CT | 26(19.7) | 32(24.1) | 1.333(0.741-2.399) | 0.925 | 0.336 |
|  | CC | 2(1.5) | 5(3.8) | 2.708(0.513-14.289) | 1.486 | 0.223 |
| Allele | T | 234(88.6) | 224(84.2) |  |  |  |
|  | C | 30(11.4) | 42(15.8) | 1.463(0.884-2.419) | 2.211 | 0.137 |
| Dominant model | TT | 104(78.8) | 96(72.2) |  |  |  |
|  | CT+CC | 28(21.2) | 37(27.8) | 1.432(0.815-2.516) | 1.562 | 0.211 |
| rs2963155 |  |  |  |  |  |  |
| Genotype | AA | 80(60.6) | 88(65.7) |  |  |  |
|  | AG | 47(35.6) | 37(27.6) | 0.716(0.423-1.212) | 1.556 | 0.212 |
|  | GG | 5(3.8) | 9(6.7) | 1.636(0.526-5.088) | 0.736 | 0.391 |
| Allele | A | 207(78.4) | 213(79.5) |  |  |  |
|  | G | 57(21.6) | 55(20.5) | 0.938(0.618-1.423) | 0.091 | 0.762 |
| Dominant model | AA | 80(60.6) | 88(65.7) |  |  |  |
|  | AG+GG | 52(39.4) | 46(34.3) | 0.804(0.488-1.325) | 0.733 | 0.392 |
| rs2963156 |  |  |  |  |  |  |
| Genotype | CC | 105(79.6) | 98(72.6) |  |  |  |
|  | CT | 25(18.9) | 32(23.7) | 1.371(0.759-2.477) | 1.101 | 0.294 |
|  | TT | 2(1.5) | 5(3.7) | 2.679(0.508-14.127) | 1.451 | 0.228 |
| Allele | C | 235(89.0) | 228(84.4) |  |  |  |
|  | T | 29(11.0) | 42(15.6) | 1.493(0.899-2.479) | 2.419 | 0.120 |
| Dominant model | CC | 105(79.5) | 98(72.6) |  |  |  |
|  | CT+TT | 27(20.5) | 37(27.4) | 1.468(0.833-2.589) | 1.770 | 0.183 |
| rs6189 |  |  |  |  |  |  |
| Genotype | CC | 132(99.2) | 134(99.3) |  |  |  |
|  | CT | 1(0.8) | 1(0.7) | 0.985(0.061-15.914) | 0.000 | 0.992 |
|  | TT | 0(0.0) | 0(0.0) |  |  |  |
| Allele | C | 265(99.6) | 269(99.6) |  |  |  |
|  | T | 1(0.4) | 1(0.4) | 0.985(0.061-15.832) | 0.000 | 0.992 |
| Dominant model | CC | 132(99.2) | 134(99.3) |  |  |  |
|  | CT+TT | 1(0.8) | 1(0.7) | 0.985(0.061-15.914) | 0.000 | 0.992 |
| rs9324924 |  |  |  |  |  |  |
| Genotype | GG | 43(32.3) | 54(40.3) |  |  |  |
|  | GT | 68(51.1) | 53(39.6) | 0.621(0.362-1.063) | 3.035 | 0.082 |
|  | TT | 22(16.6) | 27(20.1) | 0.977(0.490-1.950) | 0.004 | 0.948 |
| Allele | G | 154(57.9) | 161(60.1) |  |  |  |
|  | T | 112(42.1) | 107(39.9) | 0.914(0.647-1.290) | 0.262 | 0.609 |
| Dominant model | GG | 43(32.3) | 54(40.3) |  |  |  |
|  | GT+TT | 90(67.7) | 80(59.7) | 0.708(0.429-1.168) | 1.832 | 0.176 |
| rs7701443 |  |  |  |  |  |  |
| Genotype | GG | 54(40.6) | 48(36.1) |  |  |  |
|  | AG | 61(45.9) | 60(45.1) | 1.107(0.653-1.875) | 0.142 | 0.707 |
|  | AA | 18(13.5) | 25(18.8) | 1.563(0.761-3.210) | 1.486 | 0.223 |
| Allele | G | 169(63.5) | 156(58.7) |  |  |  |
|  | A | 97(36.5) | 110(41.3) | 1.229(0.866-1.742) | 1.336 | 0.248 |
| Dominant model | GG | 54(40.6) | 48(36.1) |  |  |  |
|  | AG+AA | 79(59.4) | 85(63.9) | 1.210(0.738-1.986) | 0.572 | 0.449 |
| rs4244032 |  |  |  |  |  |  |
| Genotype | AA | 130(97.7) | 129(95.6) | 1 |  |  |
|  | AG | 2(1.5) | 5(3.7) | 2.519(0.480-13.222) | 1.275 | 0.259 |
|  | GG | 1(0.8) | 1(0.7) | 1.008(0.062-16.285) | 0.000 | 0.996 |
| Allele | A | 262(98.5) | 263(97.4) | 1 |  |  |
|  | G | 4(1.5) | 7(2.6) | 1.743(0.504-6.026) | 0.790 | 0.374 |
| Dominant model | AA | 130(97.7) | 129(95.6) | 1 |  |  |
|  | AG+GG | 3(2.3) | 6(4.4) | 2.016(0.493-8.232) | 0.989 | 0.320 |
| rs4607376 |  |  |  |  |  |  |
| Genotype | AA | 35(26.3) | 27(20.0) | 1 |  |  |
|  | AG | 68(51.1) | 69(51.1) | 1.315(0.719-2.405) | 0.794 | 0.373 |
|  | GG | 30(22.6) | 39(28.9) | 1.685(0.844-3.366) | 2.199 | 0.138 |
| Allele | A | 138(51.9) | 123(45.6) | 1 |  |  |
|  | G | 128(48.1) | 147(54.4) | 1.288(0.918-1.809) | 2.145 | 0.143 |
| Dominant model | AA | 35(26.3) | 27(20.0) | 1 |  |  |
|  | AG+GG | 98(73.7) | 108(80.0) | 1.429(0.807-2.530) | 1.503 | 0.220 |
| rs12656106 |  |  |  |  |  |  |
| Genotype | GG | 44(33.1) | 52(38.5) | 1 |  |  |
|  | GC | 64(48.1) | 60(44.4) | 0.793(0.465-1.353) | 0.723 | 0.395 |
|  | CC | 25(18.8) | 23(17.1) | 0.778(0.3889-1.559) | 0.501 | 0.479 |
| Allele | G | 152(57.1) | 164(60.4) | 1 |  |  |
|  | C | 114(42.9) | 106(39.6) | 0.862(0.611-1.216) | 0.717 | 0.397 |
| Dominant model | GG | 44 (33.1) | 52(38.5) | 1 |  |  |
|  | GC+CC | 88(66.9) | 83(61.5) | 0.798(0.484-1.317) | 0.779 | 0.377 |
| rs12655166 |  |  |  |  |  |  |
| Genotype | TT | 98(73.7) | 106(79.1) | 1 |  |  |
|  | CT | 34(25.6) | 26(19.4) | 0.707(0.396-1.262) | 1.380 | 0.240 |
|  | CC | 1(0.8) | 2(1.5) | 1.849(0.165-20.715) | 0.256 | 0.613 |
| Allele | T | 230(86.5) | 238(88.8) | 1 |  |  |
|  | C | 36(13.5) | 30(11.2) | 0.805(0.480-1.351) | 0.675 | 0.411 |
| Dominant model | TT | 98(73.7) | 106(79.1) | 1 |  |  |
|  | CT+CC | 35(26.3) | 28(20.9) | 0.740(0.419-1.305) | 1.088 | 0.297 |
| rs12521436 |  |  |  |  |  |  |
| Genotype | GG | 50(37.9) | 57(42.2) | 1 |  |  |
|  | GA | 60(45.5) | 57(42.2) | 0.833(0.493-1.409) | 0.464 | 0.496 |
|  | AA | 22(16.6) | 21(15.6) | 0.837(0.412-1.700) | 0.242 | 0.623 |
| Allele | G | 160(60.6) | 171(63.3) | 1 |  |  |
|  | A | 104(39.4) | 99(36.7) | 0.891(0.628-1.263) | 0.421 | 0.512 |
| Dominant model | GG | 50(37.9) | 57(42.2) | 1 |  |  |
|  | GA+AA | 82(62.1) | 78(57.8) | 0.834(0.511-1.362) | 0.524 | 0.469 |

HAPE-p: high-altitude pulmonary edema patients; HAPE-r: high altitude pulmonary edema resistant (Control).

Data are shown as odds ratio (OR), 95% confidence interval (CI), and P values comparing HAPE patients and control group.
